# Supplementary material for: Cloud BioLinux: pre-configured and on-demand bioinformatics computing for the genomics community
Source: BMC Bioinformatics. 2012 Mar 19;13:42. doi: 10.1186/1471-2105-13-42 (PMC3372431; doi:10.1186/1471-2105-13-42)
Supplement: Additional file 1 — Supplementary 1 Cloud BioLinux software documentation in the form of a mini, self-contained website. Users need to download and uncompress the .zip file, and open through a web browser the "index.html" file available on the main directory. (ZIP 1823 kb). [file 1471-2105-13-42-S1.ZIP › Cloud-BioLinux-Package-Documentation/Clustering.html]

Bio-Linux Software Documentation Pages

Back to search form

### Clustering

|  |  |
| --- | --- |
| Category | Applications |
| Clustering |  |
| SubCategory | Applications |
| Graph | clmconf    clmdist    clmimac    clminfo    clmmeet    clmresidue    mcl    mcx    mcxconvert    mcxmap    mcxsubs |
| Sequences | assembly-conversion-tools    blastclust    cd-hit    clobb    gap4    gcphrap    phrap    qiime    uclust |
